# The Association Between Pathological Personality Domains and Aggravated and Risky Sexting in a Sample of Emerging Adults

**DOI:** 10.1111/sjop.70021

**Published:** 2025-08-31

**Authors:** Mara Morelli, Chiara Pazzagli, Gaetano Maria Sciabica, Antonio Chirumbolo, Silvia Andreassi

**Affiliations:** ^1^ Department of Dynamic and Clinical Psychology, and Health Studies, Faculty of Medicine and Psychology Sapienza University of Rome Rome Italy; ^2^ Department of Psychology, Faculty of Medicine and Psychology Sapienza University of Rome Rome Italy

**Keywords:** aggravated sexting, personality, PID‐5‐BF, risky sexting, sexting, young adulthood

## Abstract

Sexting is the exchange of sexually explicit content via smartphone or the Internet. These growing forms of technology‐mediated sexual behaviors are common in youth. Recent studies investigated the association between personality traits and different kinds of sexting, from normal to risky and aggressive behavior. Few studies to date have explored the role of pathological personality domains in aggravated and risky sexting in emerging adulthood, particularly within the Italian context. The main aim of this cross‐sectional study was to investigate in a sample of emerging adults the association between the five personality domains (i.e., negative affectivity, detachment, antagonism, disinhibition, and psychoticism) measured with the Personality Inventory for DSM‐5‐Brief Form (PID‐5‐BF) and aggravated and risky sexting behaviors, controlling for age and sex. The sample included 876 emerging adults (69.1% females) aged 18–29 (*M*_age_ = 23.8; SD_age_ = 2.56) recruited through snowball sampling who had completed an online survey. Participants completed the PID‐5‐BF and the subscales on aggravated and risky sexting from the Sexting Behaviors Questionnaire. The four negative binomial regression analyses showed that the two forms of aggravated sexting (i.e., sharing sexts without permission and sexting under pressure) were positively associated with detachment, antagonism, and disinhibition. Risky sexting during substance use and with strangers was negatively associated with negative affectivity and positively associated with antagonism and disinhibition. Finally, risky sexting for emotion regulation was positively associated with antagonism and disinhibition. These findings provide a broader understanding of the association between personality domains and sexting. Results have implications for further research and for implementing preventive interventions targeting emerging adults.

**Summary**

- This study addressed gaps in the literature on the association between personality domains and sexting in emerging adults.
- Detachment, antagonism, and disinhibition were positively associated with aggravated sexting.
- Antagonism and disinhibition were positively associated with risky sexting.
- Negative affectivity was negatively associated with risky sexting.
- These findings suggest that pathological personality domains may act as risk factors for unsafe and non‐consensual sexting.

## 1 Introduction

In the past decade, a growing body of literature has investigated different forms of technology‐mediated sexual behavior: one of the most prevalent is sexting (Falconer et al. 2023). Sexting refers to the exchange of provocative or sexually explicit content, also called “sexts” (such as videos, pictures, and text messages), via social media, smartphones, or the Internet (Chalfen 2009). Sexting represents a broad and complex phenomenon, encompassing different behaviors, such as sending, receiving, and consensual or non‐consensual sharing of sexual messages.

The prevalence of sexting behaviors, particularly among young people, can be attributed to the massive spread of new communication technologies and the increasing ease of access to them (Walrave et al. 2013). Studies have shown that although sexting behaviors can occur at all ages, they are more frequent in adolescents and young adults (Morelli et al. 2021; Samimi and Alderson 2014). Studies report inconsistent results regarding the phenomenon's prevalence due to varying definitions, assessment methods, and cross‐cultural differences between the countries where the studies were conducted (Morelli et al. 2021; Barrense‐Dias et al. 2017; Baumgartner et al. 2014). Recent systematic reviews and meta‐analyses (Mori et al. 2020, 2022) have shown that the prevalence of sexting behaviors in young people has significantly increased since 2009, peaked, and gradually stabilized. Furthermore, although sexting is widespread among adolescents, it appears to be more prevalent in young adults.

Several demographic variables and individual differences concur in explaining the prevalence of sexting behaviors. Among these variables are age and biological sex. Regarding age, studies suggest that sexting behaviors increase with age, from adolescence to adulthood (Mori et al. 2020; Bianchi et al. 2019; Madigan et al. 2018). Concerning biological sex, studies suggest that sexting behaviors are more prevalent in males (Dir et al. 2013; Dodaj et al. 2019; Mori et al. 2021).

The magnitude of the sexting phenomenon has led to debates about the extent to which sexting represents a risk to the health and safety of young people or whether it is a normative behavior and an expression of developmental processes instead (Döring 2014; Levine 2013). A recent categorization of sexting sheds some light on the issue, considering both potentially positive and negative aspects. Drawing on the purposes underlying sexting behaviors, and not merely their presence or absence, researchers recently categorized sexting behaviors into three dimensions: experimental, aggravated, and risky (Morelli et al. 2021; Wolak et al. 2012).

Experimental sexting refers to the consensual exchange of photos, videos, or messages with sexual content (Wolak et al. 2012). Researchers conducting studies with adolescents and young adults have shown that experimental sexting meets developmental tasks and needs, such as exploring sexuality and identity (Bianchi et al. 2019; Kosenko et al. 2017), increases self‐esteem and body image confirmation (Bianchi et al. 2017; Morelli et al. 2017), and enhances passion, intimacy, and fun with partners (Drouin and Tobin 2014; Van Ouytsel et al. 2019). They have also found that experimental sexting plays a positive role in promoting offline contact (Temple and Choi 2014) and facilitating communication among sexual minorities (Chong et al. 2015). These findings suggest that experimental sexting does not constitute dangerous or deviant behavior per se but may play a functional role in youth developmental stages (Mori et al. 2022).

Aggravated sexting refers to harmful behaviors enacted with aggressive or manipulative intent, characterized by the non‐consensual sending of sexts (Madigan et al. 2018; Wolak et al. 2012; Morelli et al. 2016a), the unauthorized dissemination of sexts and/or the exchange of sexts under threat or pressure from a partner or other individuals (Drouin and Tobin 2014; Drouin et al. 2015). Indeed, a further contribution differentiates two dimensions of aggravated sexting: perpetration (i.e., non‐consensual sexting) and victimization (i.e., receiving pressure to sext), both included under aggravated sexting (Morelli et al. 2021). Studies show that this type of sexting can expose both senders and receivers of non‐consensual sexts to harmful consequences such as cyber‐victimization (Gámez‐Guadix and Mateos‐Pérez 2019), psychological distress (Klettke et al. 2019), extortion attempts (Wolak et al. 2018), dating violence, and intimate partner aggression (Drouin et al. 2015; Morelli et al. 2016b; Van Ouytsel et al. 2021).

Risky sexting refers to sexting behaviors enacted simultaneously with behaviors considered risky for personal health and safety (e.g., sexting while using alcohol or with strangers). Numerous studies highlight a strong association between sexting, risky behaviors, and psychological distress (Mori et al. 2019). Researchers have observed the co‐occurrence of sexting with risky sexual behaviors (Houck et al. 2014), substance abuse, and depression (Morelli et al. 2016c; Benotsch et al. 2013), and suicide attempts (Dake et al. 2015). Although frequently reported in the literature, some suggest that this co‐occurrence may be misleading and could be explained by social or contextual factors such as low parental control or relationship status (Van Ouytsel et al. 2019; Temple and Choi 2014). As part of these studies, researchers also found a correlation between sexting behaviors and emotional dysregulation (Ševčíková 2016; Trub and Starks 2017), suggesting that individuals may use sexting to regulate their emotions and avoid unpleasant feelings such as boredom, sadness, and loneliness (Bianchi et al. 2019). Thus, two subtypes of behaviors fall into the category called risky sexting: one enacted in co‐occurrence with risky behaviors and one enacted as an emotion regulation strategy. The main aim of this study was to investigate, in a sample of emerging adults, the association between pathological personality domains and different forms of sexting behaviors, specifically aggravated and risky sexting, controlling for age and biological sex. These forms of sexting were chosen due to their link with adverse psychological outcomes.

Although a few studies have investigated the variables associated with different forms of sexting due to its recent categorization, recent studies show interesting results. Alongside the study of social and contextual factors, there is a growing body of studies on the role of individual personality traits as variables associated with sexting (Morelli et al. 2016c; Delevi and Weisskirch 2013), even if the psychopathological personality traits related to sexting are still understudied.

In recent years, several studies have focused on personality traits as variables associated with sexting behaviors in youth. It is possible to roughly distinguish between a strand of studies investigating the relationship between sexting behaviors and normal or nonpsychopathological personality dimensions (e.g., extraversion, neuroticism) and a still smaller strand of studies focused on personality traits considered maladaptive or psychopathological (e.g., Machiavellianism, psychopathy).

Belonging to the first group are studies that use measures of broad personality traits. They usually concern models of normal variation in personality with wide dimensions to explain differences in individual behaviors, cognition, and emotions that span the continuum from functional to dysfunctional (Mann et al. 2020). More studies belonging to this first group were mainly conducted with samples of adolescents and, more recently, with samples of young adults. The Big Five and the Hexaco model are the most widely used personality trait measures (Feher and Vernon 2021). Overall, studies have highlighted how consensual (or even experimental), non‐consensual (hence also aggravated), and risky sexting are associated with different personality traits. Specifically, non‐consensual sexting was found to be related to lower traits of conscientiousness, emotionality, and openness and higher traits of sociability (Morelli et al. 2023). Consensual sexting was found to be associated with higher traits of openness, conscientiousness, extraversion, and lower levels of self‐control (Barroso et al. 2022). Risky sexting is negatively associated with agreeableness and emotionality and positively associated with higher sociability (Morelli et al. 2023, 2020). Lastly, higher scores in honesty‐humility and conscientiousness traits were negatively associated with dimensions of sexting (Morelli et al. 2020). Finally, studies have shown that personality traits distinguish the degree of involvement in sexting behaviors (i.e., sexters vs. non‐sexters). Sexters score higher in agreeableness, conscientiousness, and openness than non‐sexters (Kokkinos et al. 2023).

Conversely, only a few studies have explored pathological personality traits in sexting so far. Data from early studies have shown inconsistent results. A study by Clancy et al. (2019) found that the Dark Triad of personality traits (i.e., psychopathy, narcissism, Machiavellianism) (Paulhus and Williams 2002) was not associated with non‐consensual sexting behaviors. Conversely, a recent study reported different results by assessing sexting as categorized in three dimensions (i.e., experimental, aggravated, and risky sexting) rather than one (Morelli et al. 2021). In a sample aged 13–30 years, researchers found that experimental consensual sexting was positively associated with Machiavellianism and narcissism.

In contrast, Machiavellianism and psychopathy showed positive associations with aggravated and risky sexting. It also emerged that age moderated the relationship between Machiavellianism and pressured sexting, stronger in younger participants, and between narcissism and non‐consensual sexting, stronger in older participants (Morelli et al. 2021). Another study found that pathological personality traits distinguish the risk of engaging in sexual‐erotic behaviors (Hernández et al. 2021). While neuroticism was not associated with such behaviors, adolescents who were more extroverted, narcissistic, uninhibited, and less empathetic faced a higher risk of victimization through sexting and online grooming.

None of these studies explored pathological personality traits related to aggravated and risky sexting in emerging adulthood, the developmental period in which sexting is most prevalent. In addition, the measures used to assess pathological personality traits are limited to specific traits with malicious, uninhibited, and narcissistic connotations and do not allow a broader assessment of the presence of both internalizing and externalizing pathological personality traits in sexting behaviors. While some of these studies were conducted in Italy (e.g., Morelli et al. 2021; Morelli et al. 2017; Morelli et al. 2020), the role of pathological personality domains remains unexplored in the Italian emerging adult population. In this regard, assessing pathological domains with the Personality Inventory for DSM‐5 (PID‐5) (Krueger et al. 2011) can significantly contribute.

The PID‐5 assesses five broad personality domains, pathological variants of the Five Factor Model (FFM) of normative personality (Hopwood et al. 2013). These domains describe an individual's main maladaptive thoughts, feelings, beliefs, and behaviors: negative affectivity (the intense and frequent experience of negative emotions and associated behaviors and manifestations, such as self‐harm and dependence, corresponding to FFM neuroticism); detachment (mistrust, withdrawal from other people and social interactions, corresponding to FFM low extraversion); antagonism (presenting behaviors that put the individual at odds with other people, lack of empathy, egocentrism, corresponding to FFM low agreeableness); disinhibition (impulsive behaviors without reflecting on possible future consequences, orientation toward immediate gratification, corresponding to FFM low conscientiousness); psychoticism (having unusual and bizarre experiences and thoughts, corresponding to FFM openness to experience) (Krueger et al. 2011, 2012; Hopwood et al. 2013). Given that sexting requires interpersonal engagement, albeit mediated through a screen, it is plausible that individuals with higher detachment may show less interest in experimental or playful sexting, which typically involves mutuality and intimacy. Instead, their engagement in sexting may be more likely to reflect manipulative or aggressive intentions.

To our knowledge, no studies have yet investigated the relationship between PID‐5 personality domains and sexting behaviors. However, research using the PID‐5 has shown that maladaptive personality traits were associated with aggressive sexual behaviors such as female coercion and sexual aggression against male victims (Russell et al. 2017), sexual violence among adult men (Russell and King 2017), asphyxiophilic fantasies (Pocknell and King 2019a), and various symptoms of sexual addiction (Pocknell and King 2019b).

From the above, it appears that studying the relationship between individual trait characteristics and sexting can help scholars better understand the function of these behaviors and pathological personality traits that could emerge as factors of vulnerability to unsafe and aggravated sexting behaviors. However, research linking sexting and maladaptive personality traits is still scarce in the literature, and, to our knowledge, no study has yet employed a comprehensive dimensional model such as PID‐5. The use of PID‐5 allows us to explore, unlike other measures previously applied in sexting research, the role of personality domains encompassing both externalizing (e.g., disinhibition) and internalizing (e.g., negative affectivity) psychopathological behaviors (Anderson et al. 2016; Cox et al. 2023). This comprehensive approach is consistent with dimensional models of psychopathology, such as the Hierarchical Taxonomy of Psychopathology (HiTOP) (Widiger et al. 2019), providing a broad and integrative framework. While models like the Dark Triad or the Dark Tetrad (Buckels et al. 2013) focus on narrower personality constructs relevant to manipulative, aggressive, and antisocial traits, they do not capture the full spectrum of maladaptive traits potentially relevant to sexting behaviors (e.g., internalizing traits). Moreover, to date, HiTOP‐specific symptom scales lack validated versions in Italian, limiting their use in our research context. Therefore, the PID‐5 offers a psychometrically validated, culturally adapted, and comprehensive tool, enabling an empirical investigation into the multifaceted personality correlates of sexting behaviors. In this sense, the present paper represents an empirical attempt to fill an important gap in the literature.

The main aim of this study was to investigate in a sample of emerging adults the extent to which the five personality trait domains measured with the PID‐5 Brief Form (i.e., negative affectivity, detachment, antagonism, disinhibition, and psychoticism) (American Psychiatric Association 2013) are associated with different forms of sexting behaviors, controlling for age and biological sex: aggravated sexting (for perpetration: sharing sexts of someone else without permission; for victimization: sexting under pressure) and risky sexting (sexting during substance and alcohol use, and/or with strangers; sexting for emotion regulation). In this study, we focused specifically on aggravated and risky sexting behaviors, which the literature highlights as more strongly linked to adverse psychological outcomes in youth. Therefore, we used only the items from the Sexting Behaviors Questionnaire (SBQ) to assess these forms.

Regarding the relationship between PID‐5 personality domains and the different kinds of sexting behaviors, we advance the following hypotheses based on the existing literature:

Hypothesis 1 *Antagonism domain scale would be associated with a higher likelihood of engaging in both aggravated and risky sexting*.

Hypothesis 2 *Disinhibition would be associated with a higher likelihood of engaging in both aggravated and risky sexting*.

Hypothesis 3 *Negative affectivity would be associated with a lower likelihood of both aggravated and risky sexting behaviors. The hypothesis is exploratory as insufficient data exist in the literature regarding the relationship between negative affectivity and both forms of sexting*.

Hypothesis 4 *Detachment would be associated with a higher likelihood of aggravated sexting behaviors but not with risky sexting behaviors. This hypothesis is exploratory, even though it rests on some considerations and research data (Hopwood et al.* 2013 *)*.

Finally, we do not make any hypotheses regarding the relationship between psychoticism and sexting behaviors because the literature is lacking.

As mentioned above, due to age and sex differences in sexting behaviors highlighted in previous research (Mori et al. 2020; Bianchi et al. 2019; Madigan et al. 2018; Dir et al. 2013; Dodaj et al. 2019; Mori et al. 2021), all the hypotheses were tested, controlling for age and biological sex.

## 2 Materials and Methods

### 2.1 Participants and Procedure

The present research involved a convenience sample of 876 Italian emerging adults (69.1% females, *n* = 605) aged 18–29 (*M*_age_ = 23.8; SD_age_ = 2.56). The sample predominantly included individuals of middle socioeconomic status. Most participants either held a high school diploma or a university degree, or were currently enrolled in higher education. Our research team initiated participant recruitment, engaging students from the psychology department of our university. Then, students shared the survey link through a snowball sampling technique among friends and acquaintances.

Additionally, posters with a QR code linking to the survey were placed in common areas of the university campus. This approach allowed us to reach a broader and more diverse sample. All participants provided informed consent by selecting the “Yes, I accept to participate in this research” option on the initial page of the online survey. The study and its procedures received approval from the ethics committee of Sapienza University of Rome (number protocol: CERT_1898DE2D3E2, 27/09/2023). They were conducted following the Ethical Principles for Medical Research Involving Human Subjects (Declaration of Helsinki).

### 2.2 Measures

#### 2.2.1 Socio‐Demographic Information

Participants were asked to report their age (in years, using an open‐ended response), biological sex (with response options: “male” and “female”), socioeconomic status (assessed with the question “What is your family's socioeconomic status?” with response options: “high”, “upper‐middle”, “middle”, “lower‐middle”, “low”, and “prefer not to say”), and educational attainment (assessed with the item “What is your highest level of education completed?” with response options: “middle school”, “high school diploma”, “bachelor's or master's degree”, and “PhD or postgraduate degree”). For statistical analyses, responses were coded numerically.

#### 2.2.2 Sexting Behaviors

Participants completed 17 items from the Sexting Behaviors Questionnaire (SBQ) (Morelli et al. 2016a). Sexting was operationally defined as sending or receiving sexually suggestive or provocative messages, photos, and/or videos via smartphone or Internet social networking sites. The SBQ was administered using a targeted selection of items measuring aggravated and risky sexting behaviors only. This selective administration aligns with the study's focus on sexting forms associated with psychological distress and maladaptive outcomes among youth. The SBQ assessed the frequency of various sexting behaviors over the past year. Consistent with the methodology employed in prior studies by Morelli et al. (2021) and by Bianchi et al. (2019), different dimensions of sexting behaviors, which exhibited good reliabilities, were examined: 10 items assessed the aggravated sexting considering dimensions of perpetration, that is (a) sharing sexts of someone else without his/her permission (named non‐consensual sexting; 6 items; a sample item is “How often have you privately sent provocative or sexually suggestive videos about someone you know without his/her consent?”; McDonald's ω of 0.94) and a victimization dimension, that is (b) sharing sexts under pressure of someone else (named sexting under pressure; 4 items; a sample item is “How often have you been pressured by a friend to send sexts?”; McDonald's ω of 0.80); (c) 4 items measured the risky sexting dimensions, which consists in sexting during substance and alcohol use and/or with strangers (named risky sexting; a sample item is “How often have you been engaged in sexting during alcohol use?” McDonald's ω of 0.70); (d) 3 items assessed being engaged in sexting for emotion regulation, that is sharing sexts when feeling lonely, bored, or in a bad mood (a sample item is “How often have you been engaged in sexting when you feel lonely?”; McDonald's ω of 0.87). Participants rated each item on the questionnaire using a 5‐point frequency scale (Likert‐type) with response options ranging from 1 (“never”), 2 (“rarely”), 3 (“2‐3 times per month”), 4 (“2‐3 times per week”), to 5 (“almost every day”). The SBQ scale scores were computed by summing the scores of the respective items.

#### 2.2.3 Maladaptive Personality Traits

The Personality Inventory for Diagnostic and Statistical Manual of Mental Disorders–5 Brief Form (PID‐5‐BF) (American Psychiatric Association 2013; Fossati et al. 2015), consisting of 25 items, assessed maladaptive personality domains, including negative affectivity (5 items; a sample item is “I worry about almost everything”; McDonald's ω is 0.67), detachment (5 items; a sample item is “I often feel like nothing I do really matters”; McDonald's ω is 0.68), antagonism (5 items; a sample item is “I often have to deal with people who are less important than me”; McDonald's ω is 0.71), disinhibition (5 items; a sample item is “I feel like I act totally on impulse”; McDonald's ω is 0.72), and psychoticism (5 items; a sample item is “I often have thoughts that make sense to me but that other people say are strange”; McDonald's ω is 0.77). Each item was rated on a 4‐point Likert scale ranging from 0 to 3, with the following anchors: 0 = “always or often false,” 1 = “sometimes or somewhat false,” 2 = “sometimes or somewhat true,” and 3 = “always or often true”. The PID‐5 BF domain scales were calculated by averaging the scores of the items within each domain.

### 2.3 Data Analysis

All investigated sexting behaviors exhibited a strongly polarized non‐normal distribution. Therefore, four negative binomial regression analyses were run in which each sexting dimension (i.e., non‐consensual sexting, sexting under pressure, risky sexting, and sexting for emotion regulation) was regressed on biological sex (0 = male; 1 = female), age (years), and the PID‐5 BF dimensions (i.e., negative affectivity, detachment, antagonism, disinhibition, and psychoticism).

In these models, estimated coefficients represent the expected change in the log count of the dependent variable for a one‐unit increase in each independent variable, holding other variables constant. Exponentiating these coefficients yields Incidence Rate Ratios (IRRs), expressed as Exp(*B*), which indicate the multiplicative change in the expected count of sexting behaviors per unit increase in the independent variable. For example, an IRR of 1.05 corresponds to an increase of 1.05 times in the expected frequency of the behavior.

The PID‐5 BF average scores were standardized (i.e., converted to z scores) before being used as independent variables in the analyses. This standardization ensures comparability between domains, which may differ in terms of scale and variance. In this study, the analyses examined the relationships between these PID‐5 BF independent variables and SBQ scores, considered as dependent variables, without making direct comparisons between the means or standard deviations of the scales.

Analyses were performed through jamovi version 2.4.11 (Jamovi 2022) and the jamovi module GAMLj3 (Gallucci 2019). There were no missing responses, as all items in the measures were mandatory for participants to complete, ensuring a complete dataset for all included variables.

## 3 Results

### 3.1 Descriptive Statistics

Descriptive statistics for the PID‐5 Brief Form dimensions were as follows: negative Affectivity (*M* = 1.53, SD = 0.63), detachment (*M* = 0.85, SD = 0.58), antagonism (*M* = 0.62, SD = 0.55), disinhibition (*M* = 0.80, SD = 0.57), and psychoticism (*M* = 0.93, SD = 0.67). Regarding SBQ dimensions, descriptive statistics were as follows: non‐consensual sexting (*M* = 6.47, SD = 2.19), sexting under pressure (*M* = 4.80, SD = 1.90), risky sexting (*M* = 5.58, SD = 2.17), and sexting for emotion regulation (*M* = 4.80, SD = 2.69).

### 3.2 Negative Binomial Regression Analyses

Concerning non‐consensual sexting, the model accounted for 14.6% of the variance, *R* ^2^ = 0.146, *χ* ^2^(7) = 63.8, *p* < 0.001. Non‐consensual sexting exhibited a positive association with detachment, *B* = 0.05, SE = 0.02, Exp(*B*) = 1.05, *p* = 0.002, antagonism, *B* = 0.04, SE = 0.01, Exp(*B*) = 1.05, *p* = 0.002, and disinhibition, *B* = 0.05, SE = 0.01, Exp(*B*) = 1.05, *p* = 0.002. Specifically, for each additional standard deviation of detachment, antagonism, and disinhibition, the frequency of non‐consensual sexting increases by 1.05 times. See Table A1 for statistics. Regarding the victimization dimension of aggravated sexting, that is, sexting under pressure, the model accounted for 12.2% of the variance, *R* ^2^ = 0.122, *χ* ^2^(7) = 60.8, *p* < 0.001. Sexting under pressure was positively related to detachment, *B* = 0.04, SE = 0.02, Exp(*B*) = 1.04, *p* = 0.03, antagonism, *B* = 0.06, SE = 0.02, Exp(*B*) = 1.07, *p* < 0.001, and disinhibition, *B* = 0.06, SE = 0.02, Exp(*B*) = 1.06, *p* = 0.001. Specifically, for each additional standard deviation of detachment, antagonism, and disinhibition, the frequency of sexting under pressure increases by 1.04, 1.07, and 1.06 times, respectively. See Table A1 for statistics.

As for risky sexting, the model explained 9.2% of the variance, *R* ^2^ = 0.092, *χ* ^2^(7) = 58.8, *p* < 0.001. Risky sexting was negatively related to negative affectivity, *B* = −0.04, SE = 0.02, Exp(*B*) = 0.96, *p* = 0.03, and positively to antagonism, *B* = 0.05, SE = 0.02, Exp(*B*) = 1.05, *p* = 0.004, and disinhibition, *B* = 0.07, SE = 0.02, Exp(*B*) = 1.07, *p* < 0.001. Specifically, for each additional standard deviation of negative affectivity, the frequency of risky sexting decreases 1.04 times; conversely, for each additional standard deviation of antagonism and disinhibition, the frequency of risky sexting increases 1.05 and 1.07 times, respectively. See Table A2 for statistics.

Lastly, concerning sexting for emotion regulation, the model accounted for 6% of the variance, *R* ^2^ = 0.060, *χ* ^2^(7) = 48.6, *p* < 0.001. Sexting for emotion regulation displayed a positive association with antagonism, *B* = 0.05, SE = 0.02, Exp(*B*) = 1.05, *p* = 0.013, and disinhibition, *B* = 0.05, SE = 0.02, Exp(*B*) = 1.05, *p* = 0.01. Specifically, for each additional standard deviation of both antagonism and disinhibition, the frequency of sexting for emotion regulation increases 1.05 times. See Table A2 for statistics.

Biological sex and age, which were included as covariates in all four negative binomial regression models, did not emerge as significantly associated factors with any of the sexting behaviors investigated.^1^

## 4 Discussion

The general aim of the present study was to explore the relationship between maladaptive personality domain measures with the PID‐5 Brief Form and aggravated and risky sexting behaviors. The five personality domains of the PID‐5 Brief Form assessed, namely negative affectivity (characterized by intense experience of negative emotions, self‐harm and dependence), detachment (social withdrawal and mistrust), antagonism (hostility, lack of empathy and egocentrism), disinhibition (impulsivity and lack of planning), and psychoticism (bizarre beliefs and perceptual experiences), capture distinct patterns of pathological personality functioning. Overall, the data show that people higher in antagonism, disinhibition, and detachment personality domains are at greater risk of engaging in aggravated and risky sexting behaviors. In contrast, participants with higher scores in negative affectivity are less likely to engage in risky sexting.

Regarding the first hypothesis on the positive relationship between antagonism and both aggravated and risky sexting behaviors, this finding is consistent with more recent studies showing that Machiavellianism and psychopathy, constructs theoretically similar to the antagonism domain in the PID‐5, were related to both aggravated and risky sexting (Morelli et al. 2021). This latter finding is, furthermore, indirectly confirmed by a study which found that higher scores in honesty‐humility traits of the Hexaco, theoretically opposite to antagonism, are negatively related to both forms of sexting (Morelli et al. 2020). Since the antagonism domain was positively associated with both aggravated and risky sexting behaviors, future studies might help investigate the moderating variables that lean more toward one type of sexting rather than the other.

Regarding the second hypothesis, analyses show that participants higher in disinhibition reported being more involved in aggravated sexting (i.e., non‐consensual sexting and sexting under pressure) and risky sexting (i.e., risky sexting during substance and alcohol use and/or with strangers and sexting for emotion regulation). This result is consistent with previous studies showing that individuals characterized by low conscientiousness, greater hedonism, low inhibition, and most in need of attention and confirmation from others were more likely to have non‐consensual and risky sexting behaviors (Barroso et al. 2022; Morelli et al. 2020). Since disinhibition is related to involvement in aggravated and risky sexting behaviors, as is the antagonism domain, future studies will have to identify which common factors of the two domains produce this effect.

Concerning the third hypothesis, regression analyses show that negative affectivity was negatively associated with risky sexting behaviors during substance and alcohol use and with strangers. However, no other significant relationships emerged between this domain and aggravated sexting behaviors or sexting for emotion regulation. As mentioned above, this hypothesis was exploratory as no studies have investigated the relationship between negative affectivity and sexting. Although our hypothesis was only partially confirmed, the results suggest that people low in emotional stability, intensely and frequently experiencing negative emotions such as guilt and shame, are significantly less likely to engage in risky sexting behaviors with strangers and those occurring alongside substance and alcohol use.

Regarding the fourth and final hypothesis, regression analyses show that detachment was associated with aggravated sexting behaviors, both non‐consensual and under pressure. Still, no relation was found with risky sexting behaviors in either form. As in the case of negative affectivity, we formulated an exploratory hypothesis, as, to our knowledge, no studies have investigated the association between detachment and sexting. However, the results suggest that individuals characterized by interpersonal withdrawal, mistrust, and low levels of extraversion are more likely to engage in aggravated sexting behaviors in both perpetration (non‐consensual sending) and victimization (exchange of sexts under pressure) dimensions, which is consistent with our hypothesis. Considering the functioning characteristics of persons falling into this profile, one could hypothesize that the mechanism underlying the risk of aggravated sexting behaviors relates to how these individuals cope with the inevitable frustration they encounter in intimate and interpersonal relationships. A dysregulation in the ability to tolerate frustration could lead to acting out by engaging in aggravated sexting behaviors. Furthermore, the finding is consistent with a recent study suggesting that individuals who are more self‐confident, able to connect with others, and adequately express emotions are more protected from the maladaptive use of sexting (Morelli et al. 2023). Although further studies are needed, our results seem to support the hypothesis that maladaptive personality domains on the internalizing side (e.g., detachment) are as much a risk factor for aggressive sexting behavior as are domains on the externalizing side (e.g., disinhibition).

Regarding the personality domain of psychoticism, no association with any form of sexting was found. As mentioned in the introduction, to our knowledge, no studies in the literature have shown significant associations between this domain and sexting behaviors. One possible explanation for this finding may be the conceptual nature of the PID‐5 psychoticism domain, which includes eccentricity, bizarre thinking, and perceptual dysregulation, traits theoretically distant from the interpersonal and emotionally charged dynamics typically involved in risky and aggravated sexting. In contrast, domains such as antagonism and disinhibition, which include traits such as manipulativeness, hostility, and impulsivity, appear more directly relevant to non‐consensual or emotionally dysregulated sexting behaviors, as our results suggest. Another possible explanation may be the brevity of the PID‐5 Brief Form (i.e., 25 items) that may have limited the instrument's sensitivity to more subtle or complex associations. Future studies using the full version of the PID‐5 (i.e., 220 items) or different personality assessment scales may help clarify the role of psychoticism in this context.

In our analyses, biological sex and age, entered as covariates in the regression models, were not found to be significantly associated with aggravated or risky sexting behaviors. This result could stem from the fact that, in our sample of emerging adults (18–29 years), maladaptive personality domains have a greater explanatory power than demographic variables. Based on previous literature highlighting the potential relevance of biological sex and age to sexting behaviors in emerging adults, these variables were included in the analyses. However, maladaptive personality domains appear more prominent in explaining these behaviors.

The lack of significant effects of sex and age could reflect that sexting behaviors and their association with personality pathology manifest similarly across genders and in this age group. Another possible explanation is that pathological personality domains represent factors more directly involved in the psychological mechanisms that drive sexting, compared to demographic characteristics. In this regard, future studies on larger and more diverse samples could investigate the potential effects of sex and age.

When interpreting our findings, it is important to remember the typical magnitude of effects in complex behavioral research. Small effect sizes are common, yet they can still reflect meaningful relationships, especially in behaviors with significant individual and social impact. While the observed Incidence Rate Ratios (IRRs) across the regression models indicate small increases in IRRs, their statistical significance suggests an effect different from zero and thus plausible to be interpreted. In fact, given the prevalence of sexting behaviors and the potential impact for a broad population, even these modest effects could have meaningful implications. This finding should be understood considering the complexity and multifactorial nature of both pathological personality traits and aggravated sexting behaviors. In the realm of deviant or risky behaviors, and particularly those influenced by underlying psychopathology, small effects are to be expected due to the many interacting individual, social, and contextual factors at play. Given that aggravated sexting represents a relatively rare but high‐risk behavior, even modest associations may have meaningful practical implications for early identification and prevention of problems, above all when they are considered together with other risk factors. Nevertheless, the practical importance should also be interpreted in light of the possible intervention's cost, feasibility, and the seriousness of the outcome.

In summary, the results of the present study show that (1) aggravated sexting, both in the aspect of perpetration and victimization, is positively associated with the maladaptive personality domains of antagonism, disinhibition, and detachment; (2) risky sexting, both that occur with substance abuse and with strangers, and sexting as emotion regulation, are positively associated with antagonism and disinhibition; (3) sexting behaviors enacted as emotion regulation are negatively associated with negative affectivity.

Despite the innovative aspects of this study, several limitations should be acknowledged. First, its cross‐sectional design prevents establishing cause‐and‐effect relationships between participants' personalities and sexting behaviors. Therefore, future research should examine how personality domains remain associated with sexting over time. Though widely used and validated, using only self‐report instruments is another limitation. It may have created a social desirability effect in participants, even more so given the study's intimate and personal subject matter, resulting in a response bias. However, using an online administration prevents this response bias by guaranteeing a sense of privacy for participants. Another limitation of the study is the sample's composition, which was predominantly female and composed of well‐educated Italian adults recruited by snowball sampling. These aspects constitute a limitation to the generalizability of our results, as gender imbalances and a relatively homogeneous educational background may affect the applicability of the results to a larger population. At the same time, snowball sampling could represent a potential sampling bias. Future research should aim to recruit more diverse and representative samples, possibly through random sampling methods by extending data collection to broader demographic, geographic, and socioeconomic groups.

## 5 Conclusion

In conclusion, the present study aimed to address some gaps in understanding the association between personality domains and sexting behaviors. Previous research (Morelli et al. 2021) suggests that underlying variables, such as individual personality traits, may explain the enactment of aggravated and risky sexting behaviors. Although there is evidence of the relationship between personality traits and different sexting behaviors, only a few studies have emphasized maladaptive personality domains, and none have been conducted so far specifically on individuals going through the developmental stage called emerging adulthood, which corresponds to the developmental stage with the highest prevalence of sexting behaviors. Moreover, a paucity of studies has investigated, both together and distinctly, the externalizing and internalizing personality dimensions. The results of the present study shed some light on these aspects, which will need to be replicated in subsequent research for confirmation. As sexting behaviors in emerging adulthood are widespread, a greater understanding of them based on underlying personality aspects helps implement prevention interventions. As this study focused on aggravated and risky sexting behaviors, and thus not on developmental and adaptive sexting behaviors, a greater understanding of the latter may allow for the implementation of interventions to promote safer and consent‐based sexual behaviors. Finally, these results can help to identify early the personality profiles of sexters who may engage in aggravated and risky forms of sexting, and to intervene promptly to prevent them from experiencing possible negative consequences related to these maladaptive behaviors.

## Author Contributions

**Mara Morelli:** conceptualization, formal analysis, methodology, project administration, writing – original draft, supervision, and writing – review and editing. **Chiara Pazzagli:** conceptualization, methodology, project administration, supervision, visualization, writing – original draft, and writing – review and editing. **Gaetano Maria Sciabica:** conceptualization, data curation, funding acquisition, investigation, writing – original draft, visualization, and validation. **Antonio Chirumbolo:** conceptualization, formal analysis, methodology, supervision, validation, and writing – review and editing. **Silvia Andreassi:** conceptualization, investigation, project administration, supervision, and writing – review and editing.

## Ethics Statement

This study was performed in line with the principles of the Declaration of Helsinki. Approval was granted by the Ethics Committee of Sapienza University of Rome [protocol ID: CERT_1898DE2D3E2, 27/09/2023].

## Conflicts of Interest

The authors declare no conflicts of interest.

## Supporting information


**Table S1:** Hierarchical negative binomial regression analyses for both dimensions of aggravated sexting.
**Table S2:** Hierarchical negative binomial regression analyses for both dimensions of risky sexting.

## Data Availability

The data that support the findings of this study are available from the corresponding author upon reasonable request.

## Endnotes

^1^
To examine the incremental contribution of biological sex, age, and personality domains on different sexting behaviors, hierarchical negative binomial regression analyses were conducted separately for each sexting behavior. In all models, biological sex was entered at Step 1, age at Step 2, and the five PID‐5 BF domains at Step 3. For non‐consensual sexting, sex explained *R* ^2^ = 0.020, age did not increase explained variance (Δ*R* ^2^ = 0.000), and the PID‐5 domains significantly improved the model, Δ*R* ^2^ = 0.126, total *R* ^2^ = 0.146. For sexting under pressure, sex explained *R* ^2^ = 0.012, age Δ*R* ^2^ = 0.000, and PID‐5 Δ*R* ^2^ = 0.110, total *R* ^2^ = 0.122. For risky sexting, sex explained *R* ^2^ = 0.010, age Δ*R* ^2^ = 0.000, and PID‐5 Δ*R* ^2^ = 0.082, total *R* ^2^ = 0.092. Finally, for sexting for emotion regulation, sex explained *R* ^2^ = 0.008, age Δ*R* ^2^ = 0.002, and PID‐5 Δ*R* ^2^ = 0.050, total *R* ^2^ = 0.060. Total *R* ^2^ values and all Exp(*B*) coefficients for each independent variable are reported in the Tables S1 and S2. These results highlight the incremental contribution of each set of independent variables.

## Appendix A

**TABLE A1:** Negative Binomial Regression Analyses for Both Dimensions of Aggravated Sexting.

	Aggravated sexting behaviors							
	Non‐consensual sexting				Sexting under pressure			
	*B*	SE	Exp(*B*)	Exp(*B*) 95% CI	*B*	SE	Exp(*B*)	Exp(*B*) 95% CI
Biological sex	−0.04	0.03	0.96	[0.91, 1.02]	−0.02	0.03	0.97	[0.91, 1.04]
Age	0.009	0.001	1.01	[0.98, 1.04]	0.01	0.02	1.01	[0.98, 1.04]
Negative affectivity	−0.02	0.02	0.98	[0.95, 1.01]	−0.03	0.02	0.97	[0.94, 1.01]
Detachment	0.05**	0.02	1.05	[1.02, 1.08]	0.04*	0.02	1.04	[1.00, 1.07]
Antagonism	0.04**	0.01	1.05	[1.02, 1.08]	0.06***	0.02	1.07	[1.03, 1.10]
Disinhibition	0.05**	0.01	1.05	[1.02, 1.08]	0.06**	0.02	1.06	[1.02, 1.09]
Psychoticism	0.01	0.02	1.01	[0.98, 1.04]	0.004	0.02	1.01	[0.97, 1.04]

*Note:* Biological sex was coded as 0 = male and 1 = female. CI = 95% confidence interval of Exp(*B*).

* *p* < 0.05.

** *p* < 0.01.

*** *p* < 0.001.

**TABLE A2:** Negative Binomial Regression Analyses for Risky Sexting and Sexting for Emotion Regulation.

	Risky sexting							
	Risky sexting				Sexting for emotion regulation			
	*B*	SE	Exp(*B*)	Exp(*B*) 95% CI	*B*	SE	Exp(*B*)	Exp(*B*) 95% CI
Biological sex	−0.02	0.03	0.98	[0.92, 1.04]	−0.06	0.04	0.94	[0.87, 1.02]
Age	0.02	0.01	1.02	[0.99, 1.05]	0.03	0.02	1.03	[0.99, 1.07]
negative affectivity	−0.04*	0.02	0.96	[0.93, 0.99]	0.00	0.02	1.00	[0.96, 1.04]
detachment	0.01	0.02	1.01	[0.98, 1.05]	0.02	0.02	1.02	[0.98, 1.06]
antagonism	0.05**	0.02	1.05	[1.02, 1.08]	0.05*	0.02	1.05	[1.01, 1.09]
disinhibition	0.07***	0.02	1.07	[1.04, 1.10]	0.05*	0.02	1.05	[1.01, 1.09]
psychoticism	0.02	0.02	1.02	[0.98, 1.06]	0.03	0.02	1.03	[0.99, 1.08]

*Note:* Biological sex was coded as 0 = male and 1 = female. CI = 95% confidence interval of Exp(*B*).

* *p* < 0.05.

** *p* < 0.01.

*** *p* < 0.001.
