# Supplementary material for: The Association Between Pathological Personality Domains and Aggravated and Risky Sexting in a Sample of Emerging Adults
Source: Scand J Psychol. 2025 Aug 31;67(1):147–57. doi: 10.1111/sjop.70021 (PMC12790106; doi:10.1111/sjop.70021)
Supplement: Supplementary file 1 — Table S1: Hierarchical negative binomial regression analyses for both dimensions of aggravated sexting. Table S2: Hierarchical negative binomial regression analyses for both dimensions of risky sexting. [file SJOP-67-147-s001.docx]

**Supplementary Materials**

**Table S.1**
**Hierarchical Negative Binomial Regression Analyses for Both Dimensions of Aggravated Sexting**

|  | Aggravated sexting behaviors | | | | | | |
| --- | --- | --- | --- | --- | --- | --- | --- |
|  | Non-consensual sexting | | |  | Sexting under pressure | | |
|  | *B(SE)* | *Exp(B)* | *Exp(B) 95% CI* |  | *B(SE)* | *Exp(B)* | *Exp(B) 95% CI* |
| Step 1 |  |  |  |  |  |  |  |
| Biological sex | -0.08** (0.03) | 0.92 | [0.87, 0.97] |  | -0.08** (0.03) | 0.92 | [0.86, 0.98] |
| R^2^ | 0.020 |  |  |  | 0.012 |  |  |
| Step 2 |  |  |  |  |  |  |  |
| Biological sex | -0.08** (0.03) | 0.92 | [0.87, 0.97] |  | -0.08* (0.03) | 0.92 | [0.86, 0.98] |
| Age | 0.007 (0.001) | 1.00 | [1.00, 099] |  | 0.001 (0.006) | 1.00 | [0.99, 1.01] |
| R^2^ | 0.020 |  |  |  | 0.012 |  |  |
| ΔR^2^ | 0.000 |  |  |  | 0.000 |  |  |
| Step 3 |  |  |  |  |  |  |  |
| Biological sex | -0.04 (0.03) | 0.96 | [0.91, 1.02] |  | -0.02 (0.03) | 0.97 | [0.91, 1.04] |
| Age | 0.009 (0.001) | 1.01 | [0.98, 1.04] |  | 0.01 (0.02) | 1.01 | [0.98, 1.04] |
| negative affectivity | -0.02(0.02) | 0.98 | [0.95, 1.01] |  | -0.03 (0.02) | 0.97 | [0.94, 1.01] |
| detachment | 0.05** (0.02) | 1.05 | [1.02, 1.08] |  | 0.04* (0.02) | 1.04 | [1.00, 1.07] |
| antagonism | 0.04** (0.01) | 1.05 | [1.02, 1.08] |  | 0.06*** (0.02) | 1.07 | [1.03, 1.10] |
| disinhibition | 0.05** (0.01) | 1.05 | [1.02, 1.08] |  | 0.06** (0.02) | 1.06 | [1.02, 1.09] |
| psychoticism | 0.01 (0.02) | 1.01 | [0.98, 1.04] |  | 0.004 (0.02) | 1.01 | [0.97, 1.04] |
| R^2^ | 0.146 |  |  |  | 0.122 |  |  |
| ΔR^2^ | 0.126 |  |  |  | 0.110 |  |  |

N*ote*. * *p* < .05; ** *p* < .01, ****p* < .001. Biological sex was coded as 0 = male and 1 = female. *CI* = 95% Confidence Interval of *Exp(B)*.

**Table S.2**
**Hierarchical Negative Binomial Regression Analyses for Both Dimensions of Risky Sexting**

|  | Risky Sexting | | | | | | |
| --- | --- | --- | --- | --- | --- | --- | --- |
|  | Risky sexting | | |  | Sexting for emotion regulation | | |
|  | *B(SE)* | *Exp(B)* | *Exp(B) 95% CI* |  | *B(SE)* | *Exp(B)* | *Exp(B) 95% CI* |
| Step 1 |  |  |  |  |  |  |  |
| Biological sex | -0.08* (0.03) | 0.92 | [0.87, 0.98] |  | -0.09* (0.04) | 0.91 | [0.84, 0.98] |
| R^2^ | 0.010 |  |  |  | 0.008 |  |  |
| Step 2 |  |  |  |  |  |  |  |
| Biological sex | -0.08* (0.03) | 0.92 | [0.87, 0.98] |  | -0.09* (0.04) | 0.92 | [0.84, 0.98] |
| Age | 0.004 (0.001) | 1.00 | [0.99, 1.01] |  | 0.01 (0.01) | 0.91 | [0.99, 1.02] |
| R^2^ | 0.010 |  |  |  | 0.010 |  |  |
| ΔR^2^ | 0.000 |  |  |  | 0.002 |  |  |
| Step 3 |  |  |  |  |  |  |  |
| Biological sex | -0.02 (0.03) | 0.98 | [0.92, 1.04] |  | -0.06 (0.04) | 0.94 | [0.87, 1.02] |
| Age | 0.02 (0.01) | 1.02 | [0.99, 1.05] |  | 0.03 (0.02) | 1.03 | [0.99, 1.07] |
| negative affectivity | -0.04* (0.02) | 0.96 | [0.93, 0.99] |  | 0.00 (0.02) | 1.00 | [0.96, 1.04] |
| detachment | 0.01 (0.02) | 1.01 | [0.98, 1.05] |  | 0.02 (0.02) | 1.02 | [0.98, 1.06] |
| antagonism | 0.05** (0.02) | 1.05 | [1.02, 1.08] |  | 0.05* (0.02) | 1.05 | [1.01, 1.09] |
| disinhibition | 0.07*** (0.02) | 1.07 | [1.04, 1.10] |  | 0.05* (0.02) | 1.05 | [1.01, 1.09] |
| psychoticism | 0.02 (0.02) | 1.02 | [0.98, 1.06] |  | 0.03 (0.02) | 1.03 | [0.99, 1.08] |
| R^2^ | 0.092 |  |  |  | 0.060 |  |  |
| ΔR^2^ | 0.082 |  |  |  | 0.050 |  |  |

N*ote*. * *p* < .05; ** *p* < .01, ****p* < .001. Biological sex was coded as 0 = male and 1 = female. *CI* = 95% Confidence Interval of *Exp(B)*.
